# Altered modulation of lamin A/C‐HDAC2 interaction and *p21* expression during oxidative stress response in HGPS

**DOI:** 10.1111/acel.12824

**Published:** 2018-08-15

**Authors:** Elisabetta Mattioli, Davide Andrenacci, Cecilia Garofalo, Sabino Prencipe, Katia Scotlandi, Daniel Remondini, Davide Gentilini, Anna Maria Di Blasio, Sergio Valente, Emanuela Scarano, Lucia Cicchilitti, Giulia Piaggio, Antonello Mai, Giovanna Lattanzi

**Affiliations:** ^1^ CNR Institute of Molecular Genetics, Unit of Bologna Bologna Italy; ^2^ Rizzoli Orthopedic Institute IRCCS Bologna Italy; ^3^ CRS Development of Biomolecular Therapies, Experimental Oncology Lab Rizzoli Institute Bologna Italy; ^4^ Department of Physics and Astronomy University of Bologna Bologna Italy; ^5^ Centre for Biomedical Research and Technologies Italian Auxologic Institute, IRCCS Milan Italy; ^6^ Department of Drug Chemistry and Technologies Pasteur Institute Italy Cenci‐Bolognetti Foundation Sapienza University of Rome Rome Italy; ^7^ Pediatric Endocrinology and Rare Diseases Unit University of Bologna Bologna Italy; ^8^ UOSD SAFU, Department of Research, Diagnosis and Innovative Technologies IRCCS - Regina Elena National Cancer Institute Rome Italy

**Keywords:** aging, *CDKN1A* (p21^WAF1/Cip1^), histone deacetylase 2 (HDAC2), Hutchinson–Gilford progeria syndrome (HGPS), lamin A/C, oxidative stress

## Abstract

Defects in stress response are main determinants of cellular senescence and organism aging. In fibroblasts from patients affected by Hutchinson–Gilford progeria, a severe *LMNA*‐linked syndrome associated with bone resorption, cardiovascular disorders, and premature aging, we found altered modulation of *CDKN1A*, encoding p21, upon oxidative stress induction, and accumulation of senescence markers during stress recovery. In this context, we unraveled a dynamic interaction of lamin A/C with HDAC2, an histone deacetylase that regulates *CDKN1A* expression. In control skin fibroblasts, lamin A/C is part of a protein complex including HDAC2 and its histone substrates; protein interaction is reduced at the onset of DNA damage response and recovered after completion of DNA repair. This interplay parallels modulation of p21 expression and global histone acetylation, and it is disrupted by *LMNA*mutations leading to progeroid phenotypes. In fact, HGPS cells show impaired lamin A/C‐HDAC2 interplay and accumulation of p21 upon stress recovery. Collectively, these results link altered physical interaction between lamin A/C and HDAC2 to cellular and organism aging. The lamin A/C‐HDAC2 complex may be a novel therapeutic target to slow down progression of progeria symptoms.

## 1 INTRODUCTION

Several evidences link lamin A/C to stress response. Prelamin A, the precursor of lamin A, is transiently accumulated during oxidative or replicative stress (Lattanzi et al., 2014; Liu, Drozdov, Shroff, Beltran, & Shanahan, 2013). Moreover, proteins involved in repair of stress‐induced DNA damage are recruited by lamins to damaged sites or inside the nuclear compartment (Gibbs‐Seymour, Markiewicz, Bekker‐Jensen, Mailand, & Hutchison, 2015; Gonzalez‐Suarez et al., 2011; Lattanzi et al., 2014). Consistent with these functions, lamin A/C has been implicated in mechanisms related to physiological (Lattanzi et al., 2014) and pathological aging (Evangelisti, Cenni, & Lattanzi, 2016), above all in progeroid laminopathies (Camozzi et al., 2014). Here, we analyzed cells from patients affected by HGPS, a premature aging syndrome linked to *LMNA* mutations, and observed an altered modulation of *CDKN1A*, encoding p21, in HGPS under oxidative stress. p21, alternatively p21^WAF1/Cip1^, is a cyclin‐dependent kinase inhibitor that targets CDK2 and CDK1 complexes and regulates cell cycle progression at G_1_/S border (Cazzalini, Scovassi, Savio, Stivala, & Prosperi, 2010). Moreover, p21 has been shown to play a role in the maintenance of G_2_‐phase arrest and to be the principal mediator of cell cycle blockade in response to DNA damage (Bell & Sharpless, 2007; Prives & Gottifredi, 2008). In fact, persistent upregulation of p21 is associated with geroconversion (Bell & Sharpless, 2007; Karimian, Ahmadi, & Yousefi, 2016; Leontieva, Demidenko, & Blagosklonny, 2015). Previous studies had shown that lamin A/C depletion is a trigger of p21 expression (Moiseeva, Bourdeau, Vernier, Dabauvalle, & Ferbeyre, 2011). Moreover, accumulation of toxic levels of prelamin A or progerin, the mutated prelamin A form found in HGPS, was associated with upregulation of p53 target genes, including *CDKN1A* (Kudlow, Stanfel, Burtner, Johnston, & Kennedy, 2008; Varela et al., 2005). However, the molecular link between lamin A/C and p21 modulation remained elusive. Thus, having observed altered p21 regulation upon oxidative stress in HGPS, we set out to identify which molecule could mediate lamin A/C effects on p21 expression. It has been demonstrated that histone deacetylase 2 (HDAC2) is involved in the regulation of *CDKN1A* gene. It was demonstrated (Peng et al., 2015) that HDAC2 is recruited to *CDKN1A* promoter by FOXO3a and regulates p21 expression in cerebellar granule neuron. Furthermore, HDAC2 has been shown to suppress p21 expression in human hepatocellular carcinoma via its binding to an Sp1‐binding site (Noh et al., 2011). On the other hand, it has been demonstrated that lamin A/C establishes direct interactions with histone deacetylases including SIRT1 (Cenni et al., 2014; Liu et al., 2012), SIRT6 (Ghosh, Liu, Wang, Hao, & Zhou, 2015), and HDAC1 (Kubben et al., 2016), while lamin partners at the nuclear envelope such as emerin, BAF, and LAP2beta interact with HDAC3 (Demmerle, Koch, & Holaska, 2013) or HDAC2 (Tsai et al., 2015). Moreover, lamin A/C has been demonstrated to bind gene promoters or neighboring domains and this binding has been linked to distinct transcriptional outcomes (Lee, Welton, Smith, & Kennedy, 2009; Lund & Collas, 2013; Mattout et al., 2011). Finally, a clear link has been established between stress‐induced chromatin remodeling, including acetylation or methylation of HDAC2 substrates H3 histone lysine 9 (H3K9) and H4 histone lysine 16 (H4K16), and lamin A/C posttranslational modifications (Ghosh et al., 2015; Lattanzi et al., 2007, 2014 ; Liu et al., 2013; Mattioli et al., 2008). Based on the whole evaluation of those reported data, we wondered if HDAC2 could mediate lamin A/C‐dependent effects on p21 expression during DDR. Our data show that lamin A/C, which binds *CDKN1A* promoter, interacts with HDAC2 to promote deacetylase activity, and the interaction is reduced at the onset of DDR and recovered after completion of DNA repair. This interplay occurring during oxidative stress response parallels modulation of p21 expression and global histone acetylation, all mechanisms disrupted by *LMNA*mutations leading to progeroid phenotypes.

## 2 RESULTS

### 2.1 Altered regulation of p21 expression during oxidative stress response in HGPS

**Figure 1:**
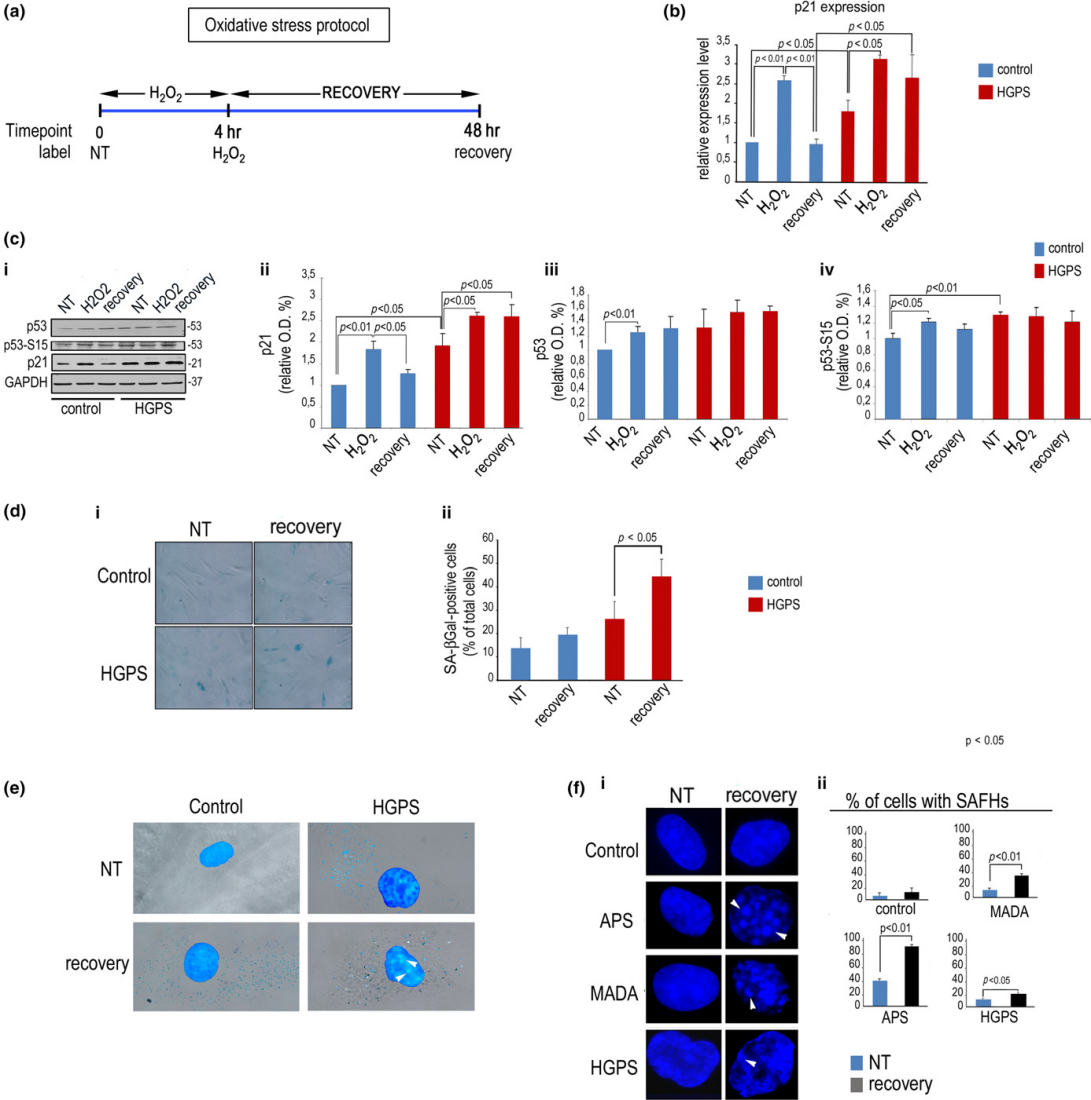
Altered p21 modulation during stress response in HGPS cells. Control or HGPS fibroblasts were left untreated (NT), exposed to H_2_O_2_ for 4 hr (H_2_O_2_), or harvested after 48 hr of H_2_O_2_ recovery (recovery). (a) Sketch of the oxidative stress experiment aligned along the time axis. (b) Quantitative RT‐PCR of CDKN1A expression. (c) (i) Western blot of p53, phospho‐p53 (p53–S15), and p21; (ii) densitometry of p21 bands, (iii) densitometry of p53 bands, and (iv) densitometry of p53–S15 bands; (d) (i) SA‐βGal staining of control and HGPS cells left untreated or after H2O2 recovery; (ii) quantitative analysis of SA‐βGal‐positive cells. (e) SA‐βGal and DAPI co‐staining in normal and HGPS cells showing SAHF (arrowheads). (f) (i) DAPI staining of nuclei of normal, APS, MADA, and HGPS fibroblasts; (ii) percentage of cells with SAHF

It has been demonstrated that HGPS fibroblasts start acquiring a senescent phenotype at late passages (Columbaro et al., 2005; Goldman et al., 2004; Meaburn et al., 2007). We hypothesized that an altered response to stress stimuli could be a major determinant of cellular aging in those cells. To test this hypothesis, we induced oxidative stress in HGPS fibroblasts and age‐ and passage‐matched controls (Table 1) and analyzed samples under basal conditions, during DNA damage response (DDR) or after 48 hr of DNA damage recovery (Figure 1a). Following 4‐hr exposure to H_2_O_2_, we did not observe lamin A/C modulation, neither in control nor in HGPS cells (Supporting Information Figure S1a,b), while prelamin A was significantly increased and its levels were decreased after stress recovery (Supporting Information Figure S1a–c), as previously reported (Lattanzi et al., 2014). Of note, also progerin, the truncated prelamin A form accumulated in HGPS cells tended to increase during DDR (Supporting Information Figure S1a–d). In this context, we evaluated the expression pattern of *CDKN1A*, whose modulation during DDR is a key event to avoid shift into a senescence program (Cazzalini et al., 2010). Modulation of *CDKN1A* levels was observed in control samples subjected to oxidative stress, where *CDKN1A* transcripts were significantly increased after 4‐hr H_2_O_2_ treatment and returned to basal level upon stress recovery (Figure 1b). However, in HGPS cells, *CDKN1A* was upregulated under basal conditions with respect to control cells, while its relative increase upon oxidative stress was lower than in controls (Figure 1b) and high transcript levels persisted after oxidative stress recovery (Figure 1b). Modulation of p21 protein level followed the same pattern (Figure 1c). These results suggested deregulation of *CDKN1A* expression. However, as proteasome‐mediated degradation of p21 is known to contribute to modulation of protein levels during DDR (Cazzalini et al., 2010), we wanted to test the possibility that proteasomal degradation of p21 could be impaired in HGPS. The same extent of proteasome‐mediated proteolysis was observed during stress recovery in control and HGPS cells, as determined by measuring protein accumulation upon MG132 treatment (Supporting Information Figure S2a). On the other hand, we did not observe any autophagic degradation of p21 during oxidative stress recovery neither in controls nor in HGPS cells, as determined by chloroquine treatment (Supporting Information Figure S2b). We concluded that p21 accumulation in HGPS cells during recovery from oxidative stress was mainly due to increase in *CDKN1A* transcripts. This could also involve a p53‐dependent mechanism, as p53 is a major player in stress response and regulator of p21 expression. However, by analyzing the stress‐response transcriptome, we found that *BRCA1*, TGF beta 1, 2, and 3, *SMAD1,* and interferon beta 1, all genes affecting p53 activity, were not dysregulated in HGPS cells (Supporting Information Figure S3). Moreover, while levels of phospho‐p53 (Serine 15, pp53) were elevated under basal conditions in HGPS fibroblasts, both p53 and pp53 dynamics during stress were comparable to age‐ and passage‐matched controls (Figure 1c). Thus, p21 increase in HGPS cells subjected to oxidative stress appeared also due to a p53‐independent dysregulation of *CDKN1A* expression.

**Table 1:** List of human dermal fibroblast cultures used in this study

Fibroblasts culture	*LMNA* mutation	Age of donor at biopsy	Gender	Passage number	Used for
CONTROL 1	None	15	F	18–22	MA,WB, IF, PLA
CONTROL 2	None	65	M	14–18	MA, IF, PLA
APS (atypical progeria syndrome)	P4R	17	F	14–15	WB, PLA
MADA (Mandibuloacral dysplasia A type)	R527H	50	M	13–14	WB, PLA
HGPS (Hutchinson–Gilford progeria syndrome)1	G608G	6	M	20–24	MA, WB, IF, PLA
HGPS (Hutchinson–Gilford progeria syndrome)2	G608G	3	F	12–14	MA
EDMD2 (Emery‐Dreifuss muscular dystrophy)	Y259D	11	F	14–16	WB, PLA

Note

IF: immunofluorescence; MA: microarray; PLA: proximity ligation assay; WB: western blot analysis.

Importantly, persistence of high p21 levels in HGPS cells was associated with cellular senescence, as demonstrated by the significant increase in the percentage of beta‐galactosidase‐positive cells observed in HGPS, but not in control cultures, upon stress recovery (Figure 1d) and by the increase in senescence‐associated heterochromatin foci (SAHF) (Aird & Zhang, 2013), which were detectable in almost all beta‐galactosidase‐positive HGPS cells (Figure 1e). The onset of a senescent phenotype upon oxidative stress recovery was also observed in cells from other progeroid laminopathies, including APS and MADA, where the low percentage of SAHF‐containing nuclei measured under basal conditions was significantly increased upon stress recovery (Figure 1f).

### 2.2 Regulation of stress response and p21 by lamin A/C

**Figure 2:**
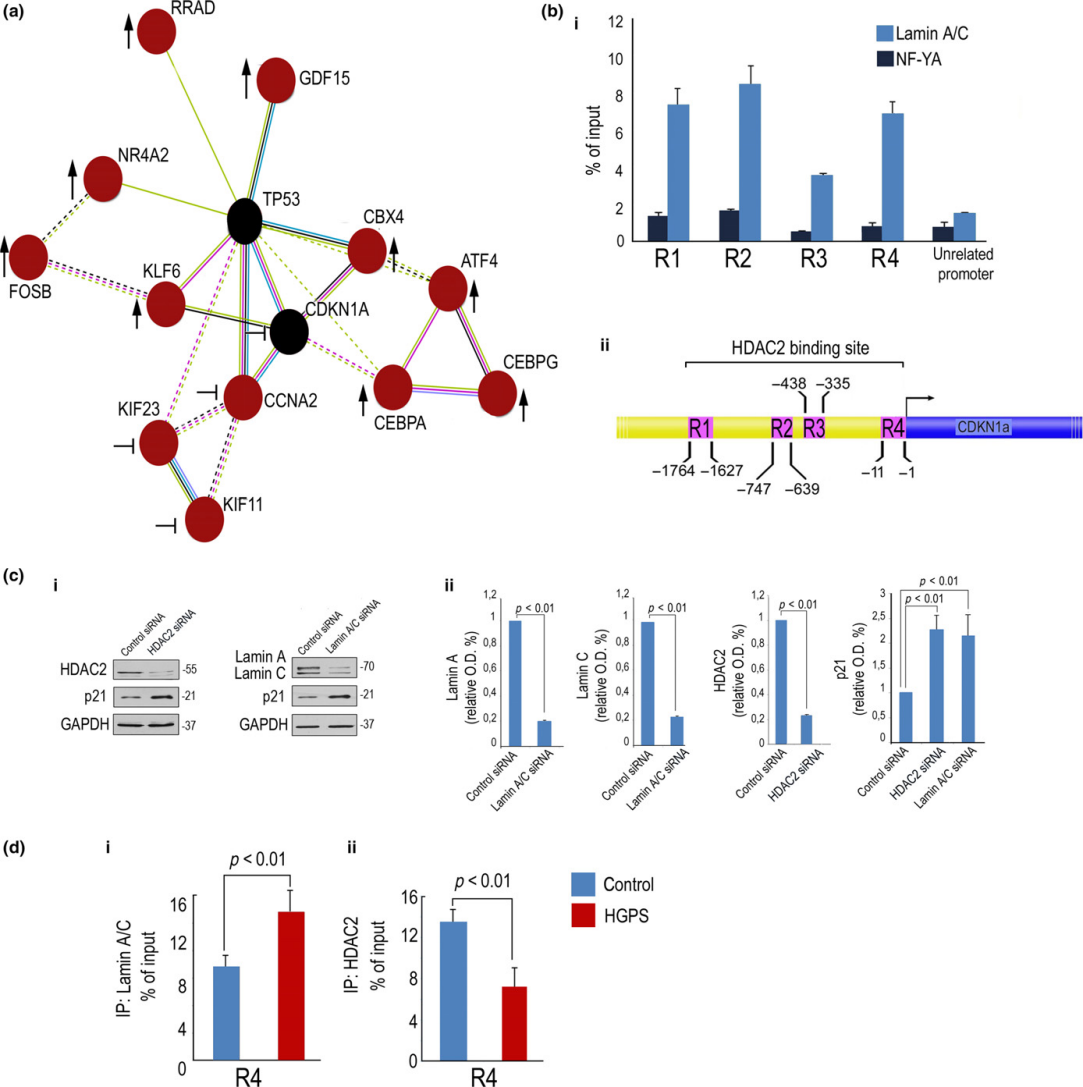
Regulation of stress response and p21 by lamin A/C. (a) String map (https://string-db.org/) indicating interconnections among genes analyzed in the microarray reported in Table 2. Genes upregulated in HGPS after H_2_O_2_ treatment with respect to H_2_O_2_‐treated human normal fibroblasts are indicated by an arrow, and genes whose regulation after H_2_O_2_ treatment is hampered in HGPS are indicated by the symbol ┤. (b) (i) Chromatin immunoprecipitation (ChIP) of lamin A/C on the *CDKN1A* promoter in normal fibroblasts. The promoter of *CDKN1A* and *CXCR4* (unrelated promoter) was detected by qPCR with specific primers listed in Experimental procedures. Protein binding is expressed as the percentage of the total DNA input. *CDKN1A* promoter regions bound by lamin A/C (R1, R2, R3, R4) are indicated; (ii) Schematic representation of regions bound by lamin A/C (R1, R2, R3, R4), on *CDKN1A* gene promoter. (c) (i) Western blot of p21, lamin A/C and HDAC2 in fibroblasts left untreated or after siRNA silencing of HDAC2 or Lamin A/C and (ii) corresponding densitometry. (d) ChIP of (i) lamin A/C or (ii) HDAC2 on the *CDKN1A* promoter in normal or HGPS fibroblasts. *CDKN1A* promoter region (R4) bound by lamin A/C or HDAC2 is indicated

However, within the small group of genes showing an altered transcriptional response to oxidative stress in HGPS cells (Table 2), the majority were interconnected in the p53‐p21 pathway (Figure 2a).

**Table 2:** Genes differently regulated upon stress in HGPS cells. Changes in gene expression in control and HGPS cells subjected to 4‐hr H_2_O_2_ treatment are reported. A ratio >1.8 or a ratio <0.55 in control or HGPS was used in the study

Gene symbol	Change in control upon stress	Change in HGPS upon stress	Gene designation	Annotated functions	**S	***L
*FOSB*	76,1	*163,8	AP−1 transcription factor subunit	Involved in cell proliferation and differentiation	X	
*GADD45A*	10,3	*17,3	Growth arrest and DNA damage‐inducible alpha	Involved in stress response	X	
*GDF15*	6,6	*17,0	Growth differentiation factor 15	Involved in stress response	X	
*SPSB1*	2,1	*6,3	SplA/ryanodine receptor domain	Probable substrate recognition component of a SCF‐like ECS		
*RRAD*	2,8	*5,9	Ras‐related glycolysis inhibitor	Interacts with CAMK2G and TPM2	X	
*SERTAD1*	2,5	*5,6	SERTA domain containing 1	Interacts with p16, CREB‐binding protein, and CDK4		
*TBX3*	3,5	*5,3	T‐box3	Regulator of developmental processes		
*PPP1R10*	1,4	*5,1	Protein phosphatase 1 regulatory subunit 10	Involved in cell cycle progression, DNA repair, and apoptosis		
*CBX4*	1,7	*5,1	Chromobox homolog 4	Epigenetic regulation of cell proliferation and differentiation	X	
*MIR320A*	1,3	*4,3	MicroRNA320a	Regulator of gene expression		
*CEBPG*	2,0	*3,8	CCAAT/enhancer binding protein gamma	Regulator of viral and cellular transcription	X	
*CEBPA*	1,1	*3,6	CCAAT/enhancer binding protein alpha	Involved in cell cycle progression	X	
*MMP25*	1,9	*3,2	Matrix metallopeptidase 25	Involved in the breakdown of extracellular matrix		
*NR4A2*	2,3	*3,0	Nuclear receptor subfamily 4 group A member 2	Regulator of gene expression	X	
*KLF6*	1,4	*3,0	Kruppel‐like factor 6	Involved in the tumor suppression	X	
*LDHC*	1,54	*2,9	Lactate dehydrogenase C	Enzyme involved in anaerobic glycolysis		
*OTUD1*	1,1	*2,8	OTU deubiquitinase 1	Deubiquitinating enzymes		
*DUSP16*	1,5	*2,4	Dual specificity phosphatase 16	Gene expression, cell proliferation, differentiation, and apoptosis		
*CSRNP2*	1,7	*2,4	Cysteine and serine‐rich nuclear protein 2	Regulator of gene expression		
*RND1*	1,1	*2,1	Rho family GTPase 1	Involved in the organization of the actin cytoskeleton		
***CDKN1A***	*4,7	2,0	Cyclin‐dependent kinase inhibitor 1	Cell cycle progression, DNA replication, and DNA damage repair	X	X
*DIP2A*	0,7	*0,5	Disco‐interacting protein 2 homolog A	Involved in axon patterning in the central nervous system		
*ASTE1*	0,8	*0,5	Asteroid homolog 1	Regulator of gene expression		
*AAK1*	0,6	*0,4	AP2‐associated kinase 1	Involved in endocytosis process		

^*^Statistically significant difference relative to corresponding untreated samples, *p* < 0.01.

^**^S, stress involvement.

***CDKN1A*** gene is the only gene in the list involved in both stress and lamin A‐related mechanisms.

^***^L, lamin interplay.

In support of a role of lamin A/C in p21 regulation, we were able to detect lamin A/C binding to *CDKN1A* promoter (Figure 2b). Specificity of lamin A/C binding was demonstrated, as an unrelated antibody, anti‐NF‐YA, did not bind the same region. As negative control, we used CXCR4 promoter that is bound neither by lamin A/C nor by NF‐YA (Figure 2b). Lamin A/C binding was detected in a region spanning 1,450 bp upstream the TSS, which includes an HDAC2 binding site (Figure 2b) (Peng et al., 2015). Consistent with a major involvement of lamin A/C and HDAC2 in p21 regulation, we observed increase in p21 protein levels in HDAC2‐depleted as well as in lamin A/C‐depleted control fibroblasts (Figure 2c). Interestingly, increased lamin A/C binding to* CDKN1A* promoter was observed in HGPS cells, but the interaction between the promoter and HDAC2 was significantly reduced (Figure 2d). The latter result suggested that loss of lamin A/C‐HDAC2 interaction in HGPS could affect HDAC2 recruitment to the p21 promoter.

### 2.3 Lamin A/C interacts with HDAC2, and binding is decreased in progeroid cells

**Figure 3:**
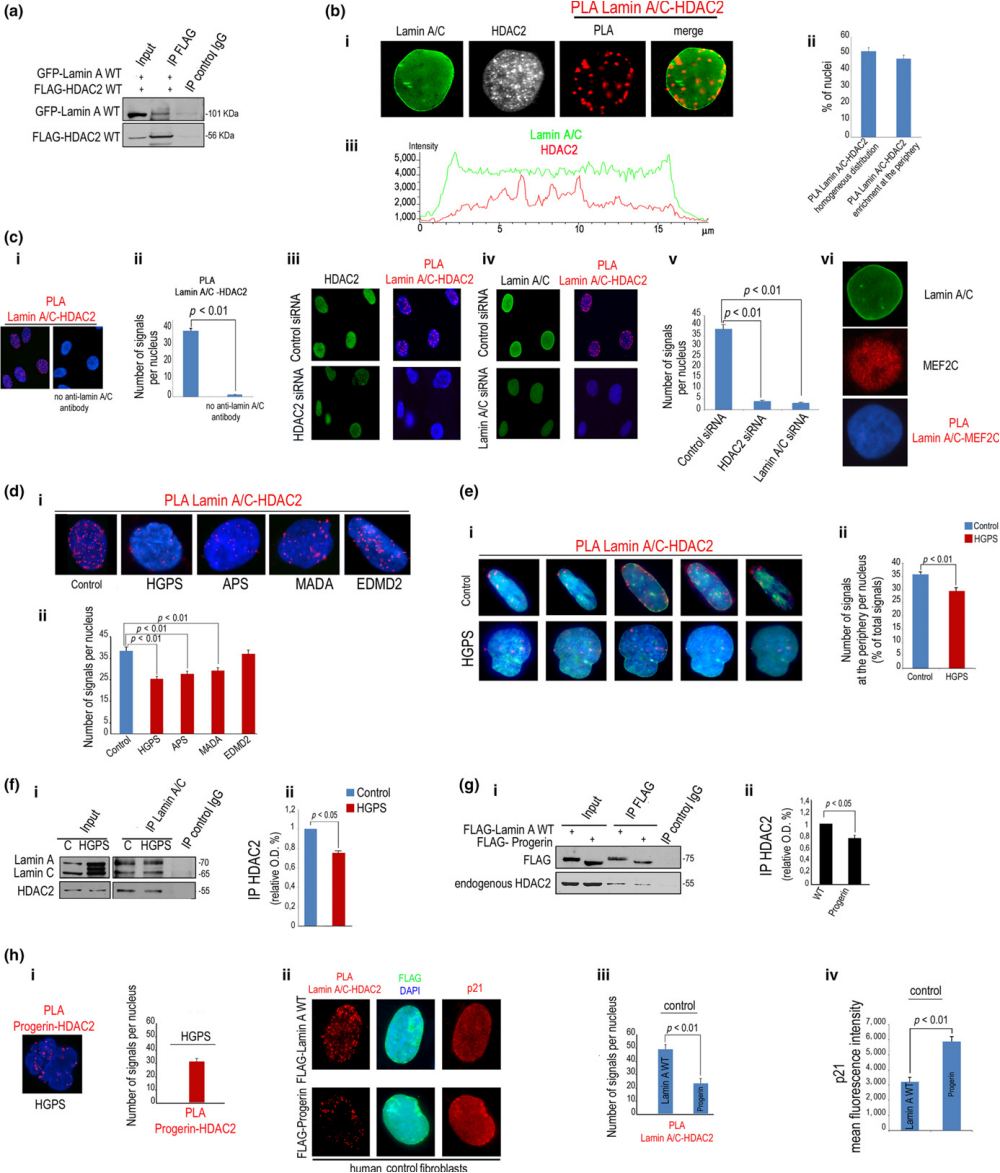
Lamin A/C interacts with HDAC2 in human normal fibroblasts more strongly than progeroid mutants. (a) Co‐IP of GFP‐lamin A and FLAG‐HDAC2 (IP FLAG) in HEK293 cells. IP control IgG, negative control. Molecular weight markers are indicated. (b) (i) Immunofluorescence staining of lamin A/C and HDAC2 and PLA of lamin A/C and HDAC2 (PLA) in human normal fibroblasts. PLA of lamin A/C and HDAC2 and lamin A/C staining are merged in the right picture (merge); (ii) percentage of nuclei showing less than 50% of signals at the periphery (PLA lamin A/C‐HDAC2 homogenous distribution) or more than 50% of signals at the periphery (PLA lamin A/C‐HDAC2 enrichment at the periphery); (iii) fluorescence intensity profile of lamin A/C and HDAC2 in a representative nucleus. (c) (i) PLA of lamin A/C and HDAC2 in the presence (left picture) or absence of lamin A/C antibody (no anti‐lamin A/C antibody) and (ii) quantitative analysis; (iii) PLA of lamin A/C and HDAC2 after HDAC2 knockdown (HDAC2 siRNA); (iv) PLA of lamin A/C and HDAC2 after lamin A/C knockdown (lamin A/C siRNA) and (v) quantitative analysis of PLA signals in the indicated samples; (vi) lamin A/C and MEF2c staining and PLA of lamin A/C and MEF2c (no signals were detected). (d) (i) PLA of lamin A/C and HDAC2 in fibroblasts from healthy subjects, HGPS, APS, MADA, or EDMD2 patients (see Table 1 for details) and (ii) quantitative analysis of PLA. (e) (i) Selected focal planes from the z‐stack of nuclei from normal or HGPS cells subjected to lamin A/C‐HDAC2 PLA. PLA signals are red dots, IF staining of lamin A/C is shown in green; (ii) number of PLA signals at the nuclear periphery reported as percentage of total PLA signals. (f) (i) Co‐IP of lamin A/C and HDAC2 in normal or HGPS fibroblasts (IP lamin A/C) and (ii) densitometric analysis of immunoprecipitated HDAC2. (g) (i) Co‐IP of FLAG‐lamin A or FLAG‐progerin and endogenous HDAC2 in HEK293 cells. IP control IgG, negative control; (ii) quantitative analysis of immunoprecipitated HDAC2, values were normalized to immunoprecipitated LMNA products. (h) (i) PLA of progerin and HDAC2 in HGPS cells and quantitative analysis of PLA signals. (ii) PLA of lamin A/C and HDAC2 in control fibroblasts transfected with WT FLAG‐lamin A or FLAG‐progerin. FLAG staining (green) is merged with DAPI. p21 co‐staining is shown on the right (p21).Representative nuclei out of 100 examined nuclei are shown; (iii) quantitative analysis of PLA signals in transfected cells performed in nuclei showing the same fluorescence intensity values for FLAG; (iv) p21 mean fluorescence intensity in transfected cells. Nuclei in c, d, e, h were counterstained with DAPI

Thus, we decided to investigate the interplay between HDAC2 and lamin A/C in control and progeroid cells. Coimmunoprecipitation (IP) experiments showed in vivo binding of lamin A and HDAC2 (Figure 3a). The interplay was confirmed by in situ proximity ligation assay (PLA) (Cenni et al., 2014) showing that HDAC2‐lamin A/C complexes were formed in the nucleus of control skin fibroblasts, and PLA signals were enriched at the nuclear envelope in 47% of cells (Figure 3b). Signals were not observed in the absence of lamin A/C antibody (Figure 3c) nor after HDAC2 or lamin A/C knockdown (Figure 3c). Moreover, an unrelated antibody (anti‐MEF2C) did not elicit any PLA signal when used in combination with anti‐lamin A/C (Figure 3c). These experiments confirmed the specificity of PLA signals. Then, we examined lamin A/C‐HDAC2 interaction in fibroblasts from progeroid laminopathies. APS, MADA, or HGPS fibroblasts expressing P4R *LMNA*, R527H *LMNA,* or G608G *LMNA,*respectively, showed significantly reduced lamin A/C‐HDAC2 interaction (Figure 3d). However, in cells from Emery‐Dreifuss muscular dystrophy (EDMD2) expressing Y259D mutated* LMNA,* HDAC2‐lamin A/C binding was comparable to controls (Figure 3d). A z‐stack analysis of PLA signal distribution showed that reduction in lamin A/C‐HDAC2 binding in HGPS occurred also at the nuclear periphery (Figure 3e). This was not due to reduced lamin A/C levels, as, in all the examined laminopathic cells, lamin A/C levels were comparable to controls (Supporting Information Figure S4a–c), although prelamin A accumulation was observed in HGPS and MADA, as previously determined (Lattanzi, 2011), and unexpectedly in APS cells (Supporting Information Figure S4a,d). Co‐IP experiments confirmed reduced binding between lamin A/C and HDAC2 in HGPS fibroblasts relative to controls (Figure 3f). However, also progerin coimmunoprecipitated endogenous HDAC2, though with lower affinity, (Figure 3g) and was detected in HDAC2‐containing complexes as determined by PLA (Figure 3h), suggesting that the mutated protein could compete for wild‐type lamin A interaction with the enzyme. In fact, overexpression of progerin in control fibroblasts exerted a dominant negative effect on lamin A/C‐HDAC2 binding (Figure 3h). Of note, in cells overexpressing progerin and showing reduced lamin A/C‐HDAC2 interaction, p21 levels were significantly increased (Figure 3h), suggesting a correlation between loss of enzyme binding to lamin A/C and increase in p21 expression.

### 2.4 Lamin A/C activates HDAC2 more strongly than progerin

**Figure 4:**
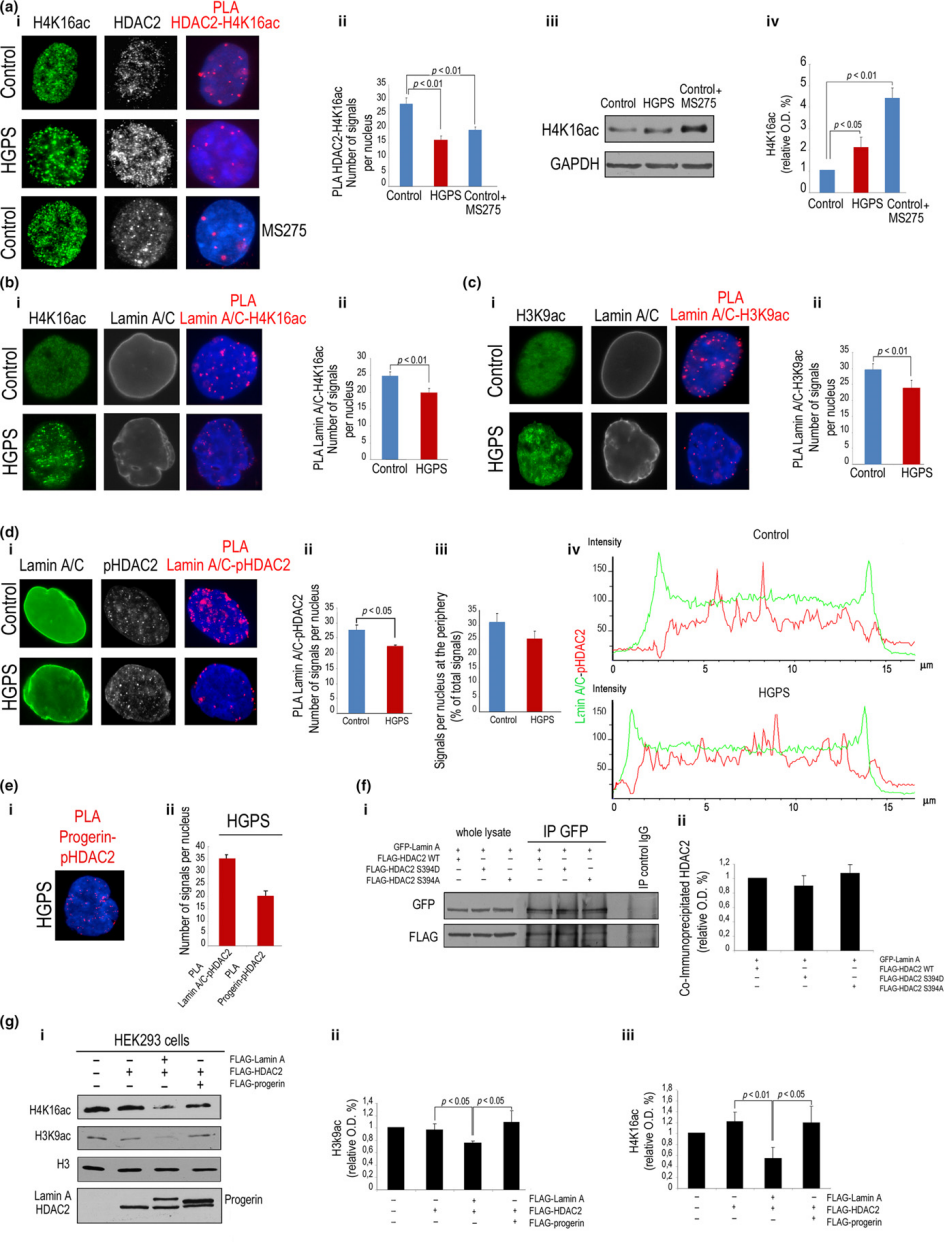
Lamin A/C complexes with acetylated HDAC2 substrates and influences HDAC2 activity. (a) (i) Human normal (control) or HGPS fibroblasts (HGPS) subjected to IF for H4K16ac and HDAC2 and PLA for HDAC2‐H4K16ac interaction. Cells treated with MS275 (MS275) were used as controls; (ii) quantitative analysis of PLA signals; (iii) western blot analysis of H4K16ac in control and HGPS cells or in control cells treated with MS275; (iv) mean densitometric values of immunoblotted H4K16ac bands. (b) (i) IF analysis of H4K16ac and lamin A/C and PLA for H4K16ac‐lamin A/C interaction in normal and HGPS cells and (ii) quantitative analysis of PLA signals. (c) (i) IF analysis of H3K9ac and lamin A/C and PLA for lamin A/C‐H3K9ac interaction and (ii) quantitative analysis of PLA signals. (d) (i) IF analysis of lamin A/C and pHDAC2 and PLA for lamin A/C‐pHDAC2 interaction in normal and HGPS cells; (ii) quantitative analysis of PLA signals and (iii) percentage of signals at the periphery with respect to total signals; (iv) fluorescence intensity profile of lamin A/C and pHDAC2 in representative nuclei of control or HGPS cells. (e) (i) PLA of progerin and pHDAC2 in HGPS cells; (ii) quantitative analysis of lamin A/C‐pHDAC2 and progerin‐pHDAC2 PLA signals in HGPS cells. (f) (i) Co‐IP of GFP‐lamin A (IP GFP) and WT FLAG‐HDAC2, S392D FLAG‐HDAC2, or S394A FLAG‐HDAC2 in transfected HEK293 cells. IP control IgG, negative control; (ii) densitometry of immunoprecipitated protein bands. (g) (i) Western blot analysis of H4K16ac and H3K9ac in non‐transfected HEK293 cells or cells overexpressing HDAC2, lamin A/HDAC2, or progerin/HDAC2 as reported in the legend; (ii) densitometry of H3K9ac and (iii) H4K16ac immunoblotted bands normalized to H3 values. Nuclei in a, b, c, d, e were counterstained with DAPI

Then, we examined lamin A/C and HDAC2 interplay with the HDAC2 substrates H4 histone acetylated on lysine 16 (acH4K16) and H3 histone acetylated on lysine 9 (acH3K9) in control and HGPS cells. As expected, HDAC2 bound acH4K16 (Figure 4a). However, the interaction was significantly reduced in HGPS, although acH4K16 levels were increased (Figure 4a). As a control, we used the HDAC2 inhibitor MS275 (Panella et al., 2016) that significantly reduced HDAC2‐acH4K16 binding, while increasing H4K16 acetylation (Figure 4a). Importantly, the HDAC2‐lamin A/C‐containing platform also included HDAC2 substrates. In fact, an interaction of lamin A/C with acH4K16 (Figure 4b) and acH3K9 (Figure 4c) was observed by PLA. In HGPS fibroblasts, we observed a significant reduction in lamin A/C binding to acetylated H4K16 (Figure 4b) and H3K9 (Figure 4c), while acetylated histone levels were increased (Figure 4b,c). As a negative control, PLA of lamin A/C and trimethylated H4K20 histone was performed (Supporting Information Figure S5).

In support of the hypothesis that lamin A/C could affect HDAC2 activity, we were able to determine an interaction between lamin A/C and the phosphorylated form of HDAC2 (serine 394, pHDAC2), which was enriched at the nuclear periphery in 33% of examined nuclei (Figure 4d). Lamin A/C interaction with pHDAC2 was significantly reduced in HGPS with respect to control cells (Figure 4d), although mean pHDAC2 fluorescence intensity and the fluorescence intensity profile were not affected (Figure 4d). On the contrary, lamin A/C staining was accumulated at the nuclear periphery in HGPS, while both peripheral and nucleoplasmic localization of lamin A/C was detected in control nuclei (Figure 4d). However, although progerin binding to pHDAC2 was also detected (Figure 4e), suggesting that progerin could compete with lamin A/C for the interaction with the active enzyme, HDAC2 phosphorylation did not influence binding affinity between the lamin A/C and HDAC2 (Figure 4f). In fact, phosphomimetic or nonphosphorylable forms of HDAC2 were equally recovered in lamin A/C‐containing immunocomplexes (Figure 4f). Importantly, data obtained in an experimental model, HEK293 cells overexpressing *LMNA* mutants, suggested that wild‐type lamin A promotes HDAC2 activity toward both acH4K16 and acH3K9, while progerin fails to properly regulate histone acetylation (Figure 4g).

### 2.5 Lamin A/C‐dependent oxidative stress response and recovery is impaired in HGPS

**Figure 5:**
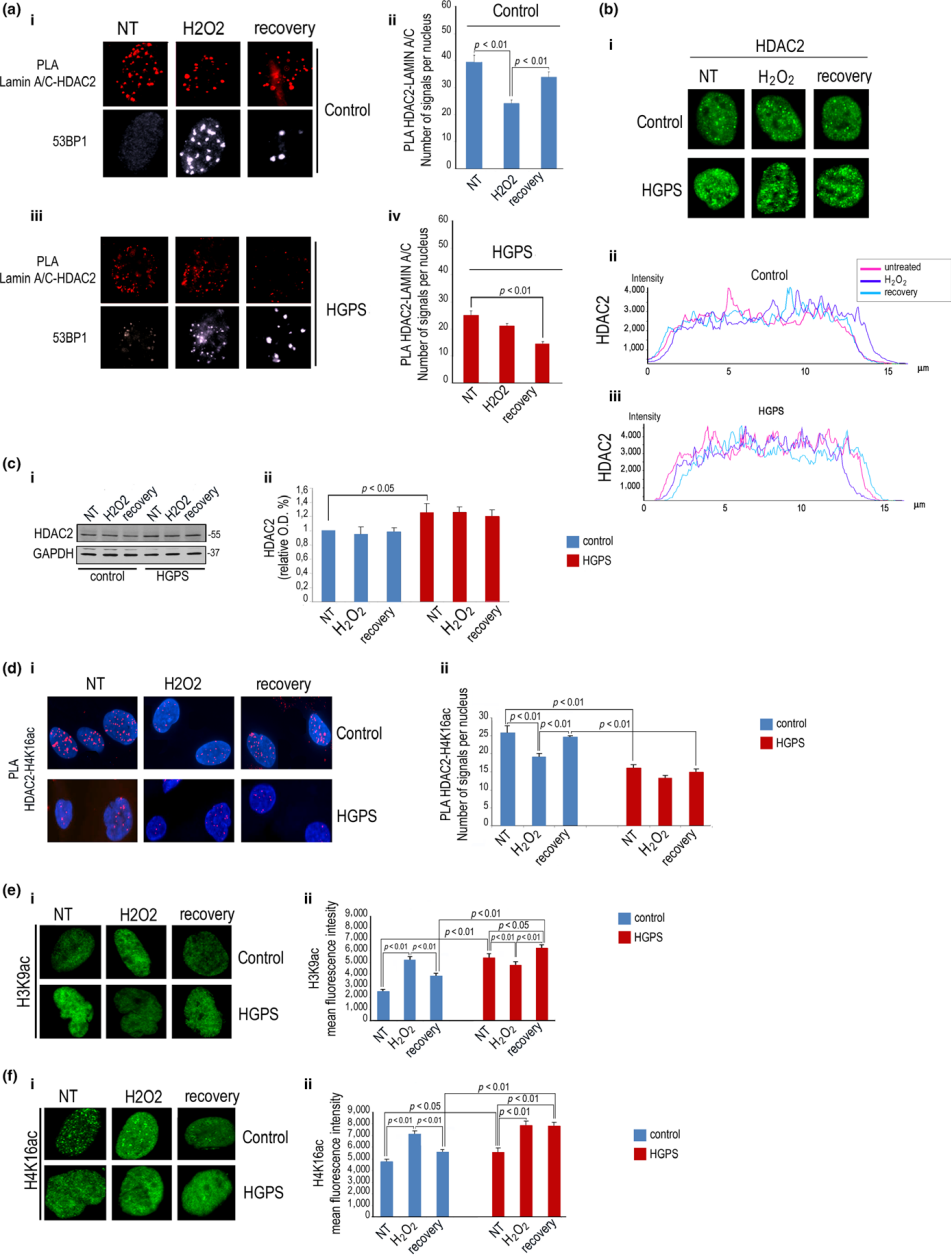
Altered modulation of lamin A/C‐HDAC2 interaction during DDR in HGPS cells. Normal (control) or HGPS fibroblasts (HGPS) were left untreated (NT), exposed to H2O2 for 4 hr (H2O2), or harvested after 48 hr of H2O2 recovery (recovery). (a) (i) PLA of lamin A/C and HDAC2 in normal human fibroblasts and (ii) quantitative analysis of PLA signals; (iii) PLA of lamin A/C and HDAC2 in HGPS cells and (iv) quantitative analysis. 53BP1 foci in (i) and (iii) are markers of DNA damage. (b) (i) IF of HDAC2 (HDAC2) in control and HGPS cells during DDR; (ii) fluorescence intensity profile of HDAC2 during DDR measured in a representative control or (iii) HGPS nucleus. (c) (i) Western blot analysis of HDAC2 in control and HGPS cells during DDR; (ii) densitometry of immunoblotted HDAC2 bands. (d) (i) PLA of HDAC2 and H4K16ac in normal and HGPS cells during DDR and (ii) quantitative analysis of PLA signals. Nuclei are counterstained with DAPI. (e) (i) H3K9ac IF staining and (ii) quantitative analysis of mean fluorescence intensity during DDR in normal or HGPS cells. (f) (i) H4K16ac IF staining and (ii) quantitative analysis of mean fluorescence intensity during DDR in normal or HGPS cells

Then, we set out to investigate the fate of lamin A/C‐HDAC2 complexes during oxidative stress‐induced DDR. In control fibroblasts, lamin A/C‐HDAC2 interaction was reduced 4 hr after oxidative stress induction, when 53 binding protein 1 (53BP1) foci indicated DNA damage, and basal levels were restored in cells that had resolved DNA damage sites (Figure 5a). Conversely, in HGPS cells, the low number of lamin A/C‐HDAC2 PLA signals observed under basal conditions was further reduced during DDR and even upon stress recovery (Figure 5a). However, HDAC2 levels and fluorescence intensity profile were not significantly affected during DDR neither in control nor in HGPS (Figure 5b‐c) although higher amount of HDAC2 was measured in HGPS cells (Figure 5c and S6). Parallel modulation of HDAC2 binding to its substrate acH4K16 was observed during oxidative stress response in control fibroblasts but not in HGPS (Figure 5d). Moreover, acetylation of H3K9 and H4K16 was increased in control fibroblasts 4 hr after oxidative stress stimulus and basal levels were restored at recovery, an acetylation pattern disrupted in HGPS cells (Figure 5e–f). These results demonstrated that defects in modulation of lamin A/C‐HDAC2 interaction alter heterochromatic H3K9 and H4K16 histone acetylation pattern and oxidative stress recovery in HGPS cells.

## 3 DISCUSSION

The main achievement of this study is the characterization of a functional interplay between lamin A/C and HDAC2 in human fibroblasts, which is modulated during DDR and contributes to regulation of HDAC2 activity and p21 expression. Importantly, we show that the protein platform, which, also includes HDAC2 substrates acetylated H3K9 and H4K16, is disrupted in HGPS, where *CDKN1A* downregulation upon stress recovery is affected leading to cellular senescence.

The observation that p21 modulation during oxidative stress response is altered in HGPS cells, both at the mRNA and protein level, suggested that functional lamin A/C was required for *CDKN1A*regulation. Our data show that in fact lamin A/C binds the *CDKN1A* promoter and the interaction between lamin A/C and HDAC2 favors HDAC2 recruitment (Noh et al., 2011), as suggested by reduced deacetylase binding in HGPS, despite increased protein levels.

Data showing that coexpression of lamin A/C and HDAC2 favors histone deacetylation, strongly suggested that lamin A/C contributes to HDAC2 activation. In support of this, we observed that deacetylation of H4K16 and H3K9 is hampered in HGPS cells and unraveled a dominant negative effect of progerin on lamin A/C‐dependent upregulation of HDAC2 activity. This dominant negative effect is associated with reduced lamin A/C‐HDAC2 interaction in cells expressing progerin, as determined both in experimental models and HGPS fibroblasts. Besides HGPS, we demonstrate a reduced interaction between lamin A/C and HDAC2 in other progeroid laminopathies. The fact that lamin A/C levels are the same in control and in progeroid cells is critical to concluding that mutated lamins disrupt stress response by competing with lamin A/C for HDAC2 interaction. Nevertheless, impaired HDAC2 recruitment to the lamin A/C‐containing platform occurs irrespective of the mutated *LMNA* sequence, a finding that suggests involvement of other molecular defects common to progeroid laminopathies, such as prelamin A accumulation (Cenni et al., 2018). However, the enhanced severity of HGPS cellular phenotype (Camozzi et al., 2014) correlated with the lower level of HDAC2‐lamin A/C interaction in HGPS with respect to other progeroid cells.

As a whole, here we characterize HGPS cells at recovery from oxidative stress, by showing that in that context lamin A/C‐HDAC2 interaction is severely affected, p21 levels are increased, and cells accumulate SAHF and senescence markers. In fact, defective modulation of p21 expression and formation of SAHF and p16 increase may account for the high number of cells that enter senescence at recovery from stress. We suggest that senescent cells accumulate in HGPS after several stress events due to failure to restore p21 levels and properly reorganize heterochromatin. It has been reported that organism senescence is related to the number of senescent cells, especially in stem cell niches (Bhatia‐Dey, Kanherkar, Stair, Makarev, & Csoka, 2016). Thus, altered HDAC2 functionality may directly contribute to the accelerated aging process in laminopathic patients.

As HDAC3 has been shown to bind the nuclear envelope protein emerin in muscle progenitors (Demmerle et al., 2013) and HDAC1‐3 have been shown to bind emerin and BAF in neuroblastoma cells (Tsai et al., 2015), we cannot rule out the possibility that tissue‐specific interactions of nuclear envelope proteins with diverse epigenetic enzymes might occur (Batrakou, Las Heras, Czapiewski, Mouras, & Schirmer, 2015; Worman & Schirmer, 2015). Along this line, mutations causing EDMD2 do not impair lamin A/C‐HDAC2 interaction (this study), while defects in emerin‐HDAC3 binding have been reported in EDMD1 (Collins, Ellis, & Holaska, 2017). Moreover, we cannot rule out that the interaction between HDAC2 and lamin A/C could be mediated by nuclear envelope proteins, other components of the protein platform or even chromatin, an issue to be further explored.

Having determined an altered interplay of lamin A/C with HDAC2 in HGPS may open new therapeutic perspectives based on the use of drugs able to restore this interplay. A number of epigenetic drugs, including TSA (Columbaro et al., 2005), MS275, and other small molecules targeting class I histone deacetylases, warrant further investigation.

Data here reported are also relevant to normal aging. Our group and others demonstrated modulation of prelamin A levels during physiological aging and particularly in response to stress stimuli (Lattanzi et al., 2014; Liu et al., 2013). It appears conceivable that prelamin A‐dependent effects on HDAC2‐lamin A/C interaction, currently under investigation, might be relevant to organism aging under physiological conditions.

It will be also interesting to determine the effect of lamin A/C‐HDAC2 interplay on other targets of HDAC2, such as regulatory microRNAs (Tian et al., 2014), nonhistone proteins, transcription factors, and enzymes, whose stability and/or activity are modulated through regulation of their acetylation levels. This will increase our knowledge of the role of lamin A/C‐containing platforms in human aging and diseases.

## 4 EXPERIMENTAL PROCEDURES

### 4.1 Cell culture and transfection

Fibroblast cultures were obtained from skin biopsies of healthy patients undergoing orthopedic surgery or laminopathic patients, following written consent. The protocol had been approved by the local ethical committee and followed EU rules. Cell cultures were established and cultured in Dulbecco's modified Eagle's medium, supplemented with 20% fetal bovine serum (Gibco) and antibiotic solution. HEK293 were cultured in D‐MEM plus 10% fetal bovine serum. Skin fibroblasts expressing P4R *LMNA* from atypical progeria syndrome (APS) (Garg et al., 2009), R527H *LMNA* from mandibuloacral dysplasia (MADA) (Novelli et al., 2002), G608G *LMNA* from HGPS (De Sandre Giovannoli et al., 2003; Eriksson et al., 2003; Pellegrini et al., 2015), and cells from Emery‐Dreifuss muscular dystrophy (EDMD2) expressing Y259D mutated *LMNA* (Mattioli et al., 2011) or control fibroblasts were included in this study (Table 1).

### 4.2 Plasmids and siRNA

Plasmids used for transfections: GFP‐lamin A, FLAG‐lamin A, FLAG‐progerin or wild‐type FLAG‐HDAC2, nonphosphorylable FLAG‐HDAC2 S394A, and phosphomimetic FLAG‐HDAC2 S394D (Peng et al., 2015). Transfection of HEK293 cells was performed using FuGENE 6 (Promega) according to the manufacturer's instructions, and cells were incubated for 24 hr after transfection. Transfection of human fibroblasts was performed using AMAXA Nucleofector (Lonza), according to the manufacturer's instructions. After transfection, fibroblasts were incubated for 48 hr. Expression of HDAC2 or lamin A/C was silenced in human fibroblasts with predesigned siRNA: HDAC2 siRNA (SC‐29345, Santa Cruz) and lamin A/C siRNA (SC‐3577 Santa Cruz) according to the manufacturer's instructions. 50 pmols of siRNA were used for transfections. HDAC2 silencing was performed for 7 days, and lamin A/C silencing was performed for 12 days. Validation was performed by western blot.

### 4.3 Microarray hybridization, processing, and data analysis

Gene expression profile was evaluated using HumanHT‐12 v3 BeadChips whole‐genome hybridization assay (Illumina), which is based upon fluorescence detection of biotin‐labeled cRNA. Each array contains full‐length 50‐mer probes representing >48,000 well‐annotated RefSeq transcripts, including >25,400 unique, curated, and up‐to‐date genes derived from the National Centre for Biotechnology Information Reference Sequence database (NCBI). Initially, 300 ng of total RNA was converted to cDNA, followed by an in vitro transcription step to generate Biotin‐16‐UTP‐labeled cRNA using the Ambion Illumina Total Prep RNA Amplification Kit (Ambion). The labeled probes were mixed with hybridization reagents and hybridized overnight to the HumanHT‐12 v3 BeadChips (Illumina). Following washing and staining, the BeadChips were imaged using the Illumina BeadArray Reader (Illumina) to measure fluorescence intensity for each probe. The intensity of the signal corresponds to the quantity of the respective mRNA in the original sample. Raw data were extracted using GenomeStudio software (Illumina); then, all samples were normalized by quantile normalization procedure. Statistical comparisons were performed by Student's *t* test: To define a statistically significant variation in expression level, we considered a combined threshold based on p‐value (*p* < 0.01) and ratio between average gene expression values (*R* > 1.8 or *R* < 0.55).

### 4.4 Antibodies and drugs

Antibodies used in this study are listed in Table 3. Anti‐lamin A/C and anti‐prelamin A antibodies used in this study have been previously characterized in control and HGPS cells (Cenni et al., 2014; Columbaro et al., 2005; Lattanzi et al., 2014). The class I HDAC inhibitor MS‐275 (entinostat) was applied 5 µM for 18 hr to fibroblasts cultures. To induce oxidative stress, 100 µM hydrogen peroxide (H_2_O_2_) was added to human fibroblasts cultures for 4 hr. Recovery was measured 48 hr after H_2_O_2_ removal. After 24 hr of H_2_O_2_ removal, 40 µM MG132 (Sigma) was applied for 4 hr to fibroblasts cultures, whereas 1 µM chloroquine (Sigma) was applied for 20 hr.

**Table 3:** List of antibodies used in this study

ANTIBODY	Code	Species	IF dilution	WB dilution	PLA dilution	ChIP	IP
anti‐lamin A/C	Santa Cruz, SC‐6215 (*N*‐terminus)	Goat polyclonal	1:100	1:100	1:100	4 µg	
anti‐lamin A/C	Novocastra (C‐terminus)	Mouse monoclonal					4 µg
anti‐prelamin A	Santa Cruz SC‐6214 (amino acids 644‐664)	Goat polyclonal		1:100			
anti‐HDAC2	Abcam, AB227149	Rabbit polyclonal	1:200	1:2000	1:200	5 µg	
anti‐HDAC2	Santa Cruz, SC‐55541	Mouse monoclonal	1:200		1:200		
anti‐phopsho‐HDAC2 S394	Abcam, AB75602	Rabbit polyclonal	1:50		1:50		
antiprogerin	Enzo 13A4	Mouse monoclonal	1:50	1:100	1:10		
anti‐p16^ink4^	Santa Cruz sc‐468	Rabbit polyclonal		1:100			
anti‐H4K16 acetylated	Abcam, ab109463	Rabbit polyclonal	1:200	1:4,000	1:200		
ant‐H4K20 acetylated	Upstate 07463	Rabbit polyclonal	1:200		1:200		
anti‐H3K9 acetylated	Millipore, 06942	Rabbit polyclonal	1:200	1:500	1:200		
anti‐H3	Santa Cruz	Goat polyclonal		1:500			
anti‐p21	Invitrogen, MA5‐14949	Rabbit polyclonal	1:100	1:1,000			
anti‐Flag tag	Sigma	Mouse monoclonal	1:300	1:3,000			1 µg
anti‐GFP tag	Santa Cruz	Rabbit polyclonal		1:500			3 µg
anti‐GAPDH	Millipore	Mouse monoclonal		1:10,000			
anti−53BP1	Cell signaling 4937S	Rabbit polyclonal	1:50				
anti‐p53	Santa Cruz sc‐126	Mouse monoclonal		1:500			
anti‐p53‐S15	Cell signaling sc‐9284	Rabbit polyclonal		1:500			
anti‐NF‐YA	Santa Cruz	Rabbit polyclonal				4 µg	
anti‐MEF2C	Cell signaling sc‐9792	Rabbit polyclonal			1:200		

Note

ChIP: chromatin immunoprecipitation assay; IF: immunofluorescence; IP: immunoprecipitation: PLA: proximity ligation assay; WB: western blot analysis.

### 4.5 In situ PLA

In situ PLA was performed using kits from Sigma‐Aldrich: Duolink^®^ In Situ Detection Reagents Orange (DUO92007) according to the manufacturer. Briefly, methanol‐fixed samples were incubated with 4% BSA in PBS to saturate nonspecific binding and subsequently with primary antibodies overnight at 4°C. Thereafter, slides were incubated for 1 hr at 37°C with secondary probes diluted to final concentrations of 1:5. Ligation solution was added for 30 min at 37°C. Ligation solution was removed with wash buffer A, and amplification solution was added for 100 min at 37°C and removed with wash buffer B. Duolink in situ mounting medium with DAPI was added, and samples were observed by a Nikon Eclipse Ni fluorescence microscope equipped with a digital CCD camera and NIS‐Elements AR 4.3 software. Quantitative analysis of PLA results was performed using Duolink Image Tool software (Sigma) by counting 300 nuclei per sample.

### 4.6 Immunofluorescence analysis

Cells grown on coverslips were fixed with absolute methanol at −20°C for 7 min. After saturation with 4% BSA in PBS, coverslips were incubated with primary antibodies overnight at 4°C and with secondary antibodies for 1 hr at RT. Samples mounted with antifade reagent were observed with a Nikon Eclipse Ni epifluorescence microscope. Images captured with NIS‐Elements 4.3 AR software were elaborated using Photoshop CS.

### 4.7 Immunoblot and IP

For western blot analysis, human fibroblasts were lysed in a buffer containing: 20 mM Tris‐HCl (pH = 7.5), 1% SDS, 1 mM Na3VO4, 1 mM PMSF, 5% beta‐mercaptoethanol, and protease inhibitors. After sonication (ultrasonic frequency 30 kHz), centrifugation, and protein quantification by Bradford method, proteins were subjected to SDS gradient gel (5%–20%) electrophoresis and transferred to nitrocellulose membrane overnight at 4°C. Incubation with primary and secondary antibodies was performed, and immunoblotted bands were revealed by Invitrogen ECL detection system. Densitometry was performed by a Bio‐Rad GS800 Densitometer. Densitometric values were normalized to corresponding GAPDH bands if not differently stated.

For IP, transfected cells were lysed in a high salt and high detergent‐IP buffer containing: 50 mM Tris‐HCl (pH = 8), 300 mM NaCl, 0.1% SDS, 1% NP‐40, 1 mM PMSF, and protease and phosphatase inhibitors. For each sample, 700 µg of lysate was incubated overnight with 1 µg of anti‐FLAG or 3 µg of anti‐GFP or nonspecific immunoglobulins form Santa Cruz as a negative control. After the addition of 30 μl of protein A/G (Santa Cruz) for 60 min at 4°C, the immunoprecipitated proteins were washed 3 times in IP buffer. Later, the samples were added to Laemmli's buffer, boiled, and subjected to western blot analysis.

IP experiments were also performed to detect endogenous protein in human dermal fibroblasts. To obtain an enriched nuclear fraction, cell pellet was resuspended in a buffer containing 10 mM Tris‐HCl pH 7.8 and protease inhibitors for 10 min on ice to induce hypotonic shock. Cells were sheared by passages through a 22‐gauge needle, and nuclei were recovered by 1,000 *g* centrifugation at 4°C for 10 min. High detergent‐IP buffer containing 50 mM Tris‐HCl (pH = 8), 150 mM NaCl, 0.1% SDS, 1% NP‐40, 1 mM PMSF, and protease and phosphatase inhibitors was added to pellet of enriched nuclear fraction. This IP buffer has been previously shown to solubilize hard‐to‐extract nuclear lamina constituents. Then, about 350 µg of protein nuclear extracts was immunoprecipitated with 4 µg of anti‐lamin A/C antibody overnight at 4°C. Control IPs were performed in the presence of nonspecific immunoglobulins. After addition of 30 µl of Protein A/G, lysates were incubated at 4°C for 1 hr and samples were washed 3 times in IP buffer. Immunoprecipitated protein complexes were added to Laemmli's buffer, boiled, and subjected to western blot analysis.

### 4.8 Quantitative RT‐PCR

Total RNA was extracted using the TRI Reagent Solution (Ambion) and treated with TURBO DNase (Ambion). cDNAs were prepared by reverse transcription using the High‐Capacity RNA‐to‐cDNA Kit (Applied Biosystems) according to the manufacture's protocol. Gene expression was quantified by qPCR using Fast SYBR Green PCR Master Mix (Applied Biosystems) and analyzed by StepOnePlus Real‐Time PCR System (Applied Biosystems). The following primers were used: GAPDH, 5′‐TCGGAGTCAACGGATTTGGT‐3′ (forward) and 5′‐TTGCCATGGGTGGAATCATA‐3′ (reverse); CDKN1a 5′‐GCAGACCAGCATGACAGATTT‐3′ (forward) and 5′‐GGATTAGGGCTTCCTCTTGGA‐3′ (reverse). *CDKN1a* transcript level was normalized to those of the internal standard gene *GAPDH*. The ΔΔCt method was used to measure the fold change of expression levels. Dissociation curve analysis was performed to confirm that the fluorescence was derived from specific amplification.

### 4.9 Chromatin IP

1% formaldehyde was added directly to SW‐480 cells, and cells were incubated at 22°C for 10 min. The reaction was stopped adding 0.125 m glycine. Then, cells were rinsed with cold 1× PBS, incubated with 0.2 × trypsin‐EDTA in 1× PBS, and scraped. Cells were centrifuged, washed in cold 1× PBS plus 0.5 mm PMSF, and resuspended in lysis buffer (5 mm piperazine N,N bis zethone sulfonic acid (pH 8.85) mm KCl, 0.5% Nonidet P‐40). Next, nuclei were solicited in the sonication buffer (0.1% SDS, 10 mm EDTA, 50 mm Tris‐HCl (pH 8), and 0.5% deoxycholic acid) for 10 min using a microultrasonic cell disruptor. The chromatin was sheared to an average size of 500 base pairs, and IP was performed with protein G‐agarose (KPL). The chromatin solution was precleared by adding protein G for 1 hr at 4°C and incubated at 4°C overnight with 4 μg of antibody or nonspecific immunoglobulins (IgGs, Santa Cruz) as negative control. Input was collected from a control sample supernatant (not immunoprecipitated antibody). Immunoprecipitates were recovered by incubation for 2 hr at 4°C with protein G‐agarose precleared previously in IP buffer (1 μg/μl bovine serum albumin, 1 μg/μl salmon testis DNA, protease inhibitors, and PMSF). Reversal of formaldehyde cross‐linking, RNase A, and proteinase K treatments was performed. DNA was phenol‐extracted, ethanol‐precipitated, and analyzed by PCR. DNA representing 0.005%–0.01% of the total chromatin sample (input) or 1%–10% of the immunoprecipitated was amplified using the following pair of primers: *CDKN1a*R1: forward 5′‐ TCATTGTGAAGCTCAGTACCAC ‐3′; reverse 5′‐ CCTTGAAGCCCCTCTGCTTT‐3′; *CDKN1a*R2: forward 5′‐ AGGTGAGTGTAGGGTGTAGGG‐3′, reverse‐ 5′ TTCCGGGAAGGAGGGAATTG‐ 3′; *CDKN1a*R3 forward 5′‐ TGCAGAGAGGTGCATCGTTT ‐3′; reverse‐ 5′‐ CACTCTGGCAGGCAAGGATT ‐ 3′, PCR was performed with the following pair of primers: *CDKN1a*R4: forward 5′CTCTGTTCTGTCTGCCTTGCT‐3′, reverse 5′‐TGGTCCTAGCTCTGCCAGTTA‐3′. *CXCR4* promoter (unrelated promoter): forward 5 AGTGGTTTGACCTCCCCTTT ‐3′; reverse‐ 5‐ ACTTGCACCTGCCAGTCTTC ‐ 3′.

Antibodies used for ChIP are listed in Table 3 (Athar & Parnaik, 2015). Quantitative PCR (qPCR) was performed using SYBR Green on an ABI Prism 7,500 apparatus (Applied Biosystems). The percentage (Input %) value was calculated as follows: Input % = 100/2 ΔCt [normalized ChIP]. The “Input %” value represents the enrichment of NF‐YA and lamin A/C on a specific region of the *CDKN1A* promoter.

### 4.10 SA‐βGal assay

SA‐βGal staining on human fibroblasts was performed using the Senescence Detection kit (Abnova) according to the manufacturer's instructions. Cells were then incubated with SA‐βGal solution for 16 hr at 37°C. Results are reported as percentage of counted cells.

### 4.11 Statistical analysis

Graphs in each panel represent mean values from at least three independent experiments ± standard error for PLA assay and IF or ± standard deviation for WB and PCR. Statistically significant differences (*p* < 0.05) are calculated by Student's *t* test.

## CONFLICTS OF INTEREST

None declared.

## AUTHOR CONTRIBUTIONS

Elisabetta Mattioli designed the study and performed PLA, co‐IP, immunofluorescence, western blot experiments, and senescence tests. *Davide Andrenacci* designed the study and performed all qPCR studies and statistical evaluation of results. Lucia Cicchilitti, Cecilia Garofalo, Giulia Piaggio, and Katia Scotlandi designed and performed lamin A/C ChIP studies and statistical analysis and interpreted the results. Sabino Prencipe performed PLA studies in laminopathic cells. Daniel Remondini, Davide Gentilini, and Anna Maria Di Blasio performed the microarray analysis, interpretation, and statistical evaluation of results. Emanuela Scarano provided APS biopsies and performed diagnosis of APS. Sergio Valente and Antonello Mai designed HDAC inhibitor studies and interpreted results. Giovanna Lattanzi designed the study, interpreted results, and prepared the manuscript.
