# Corrigendum

**DOI:** 10.1111/acel.12885

**Published:** 2018-11-28

Mattioli E, Andrenacci D, Garofalo C, et al. Altered modulation of lamin A/C‐HDAC2 interaction and p21 expression during oxidative stress response in HGPS. *Aging Cell*. 2018;17:e12824. https://doi.org/10.1111/acel.12824

In the article “Altered modulation of lamin A/C‐HDAC2 interaction and p21 expression during oxidative stress response in HGPS,” the author (Cicchillitti) would like to correct her last name and add her current address:

Lucia Cicchillitti

Present Address: Gynecologic Oncology Unit, Department of Experimental Clinical Oncology, IRCCS ‐ Regina Elena National Cancer Institute, Rome, Italy.

The author would like to apologize for the inconvenience caused.

