# Supplementary figures and images for: Altered modulation of lamin A/C‐HDAC2 interaction and p21 expression during oxidative stress response in HGPS
Source: Aging Cell. 2018 Aug 15;17(5):e12824. doi: 10.1111/acel.12824 (PMC6156291; doi:10.1111/acel.12824)

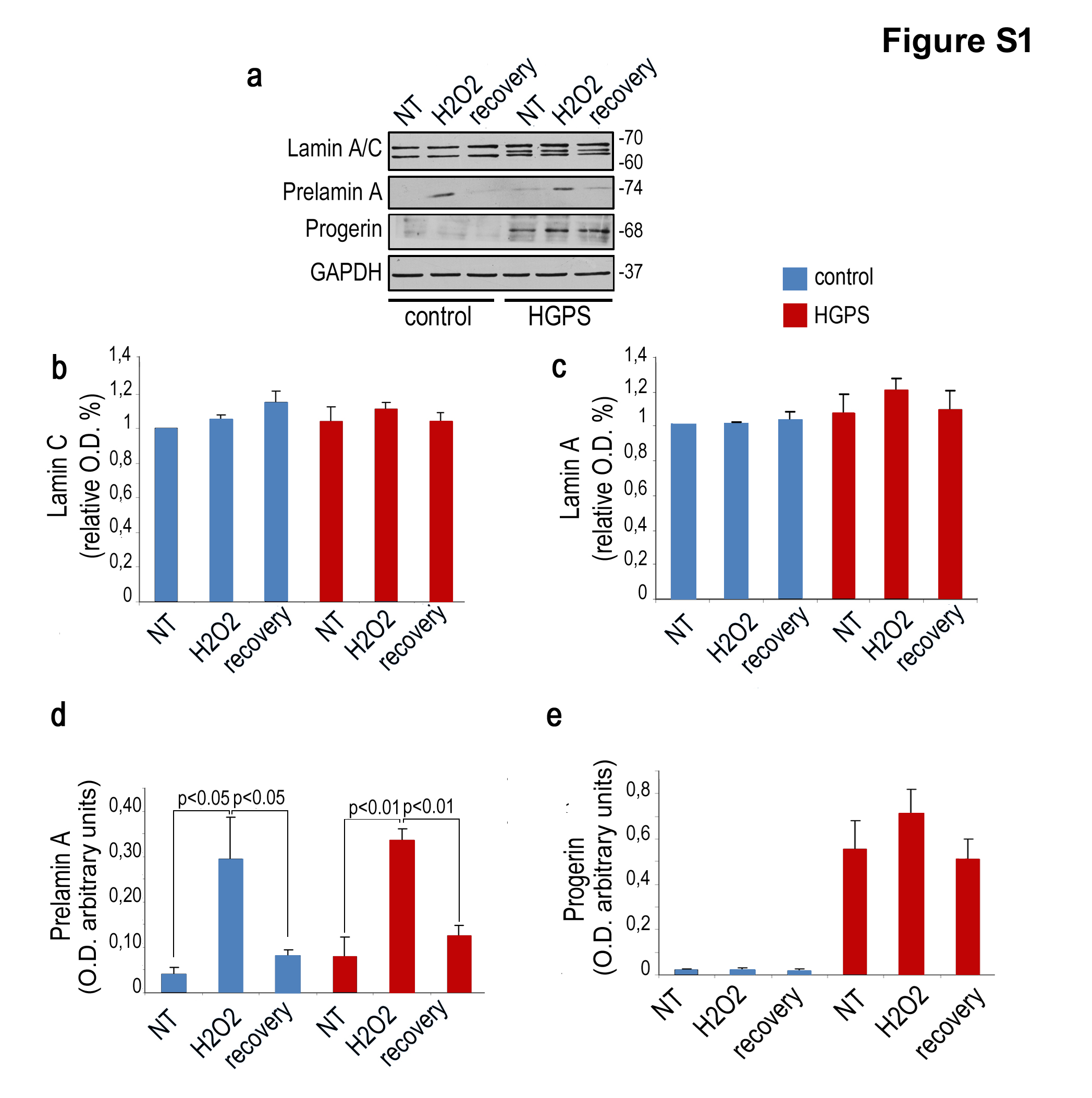

Supplement: Supplementary file 1 [file ACEL-17-e12824-s001.tif]

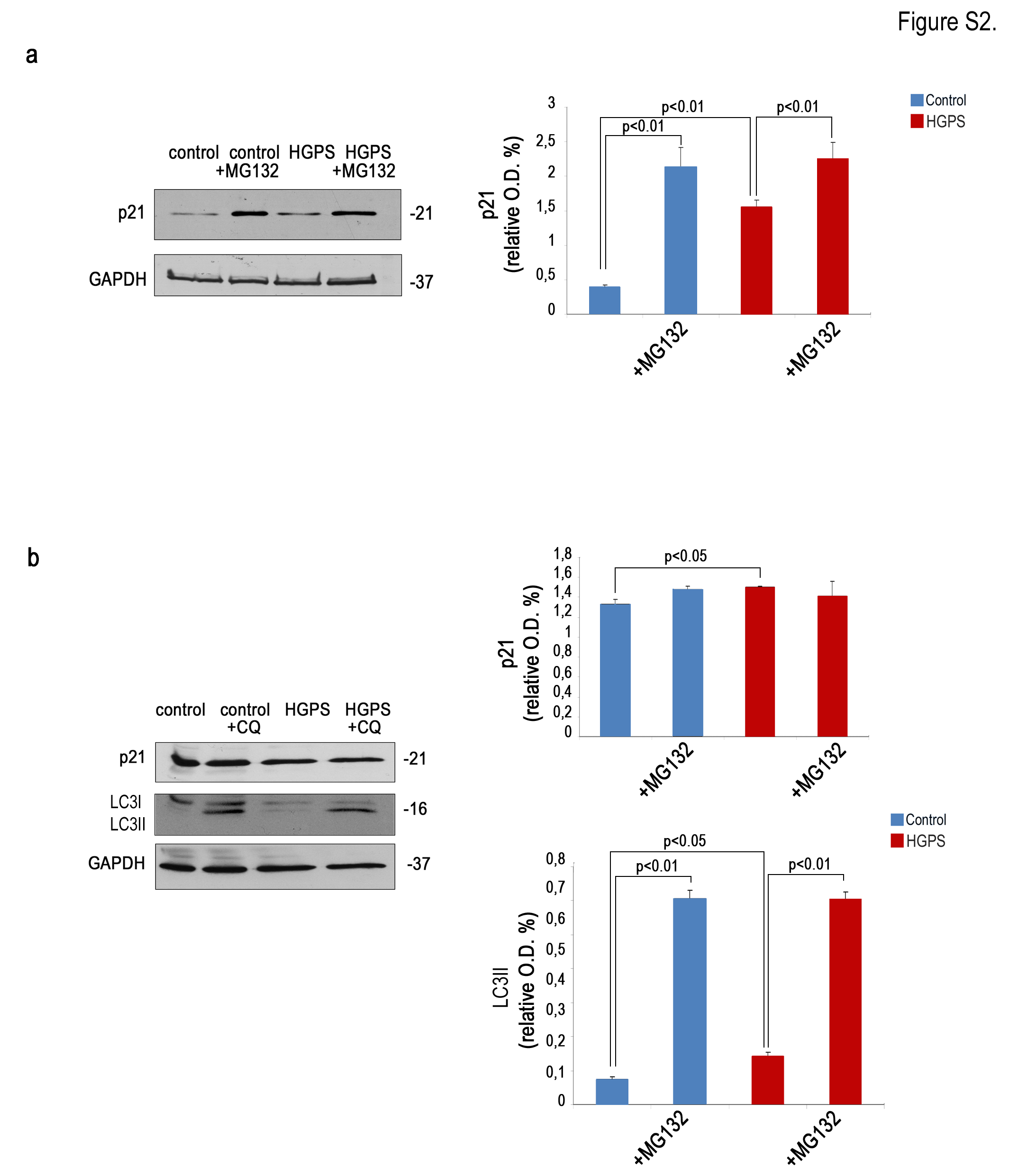

Supplement: Supplementary file 2 [file ACEL-17-e12824-s002.tif]

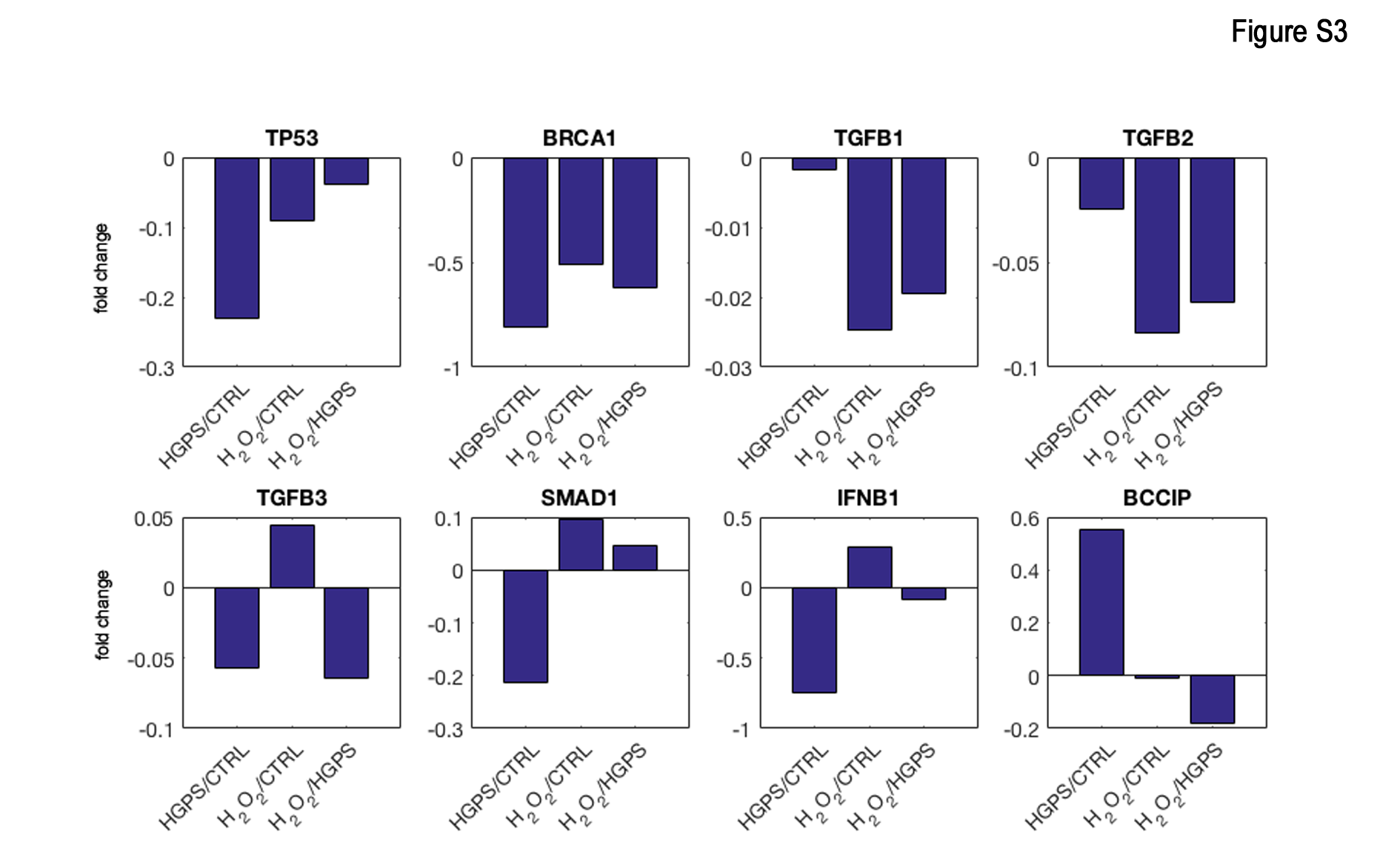

Supplement: Supplementary file 3 [file ACEL-17-e12824-s003.tif]

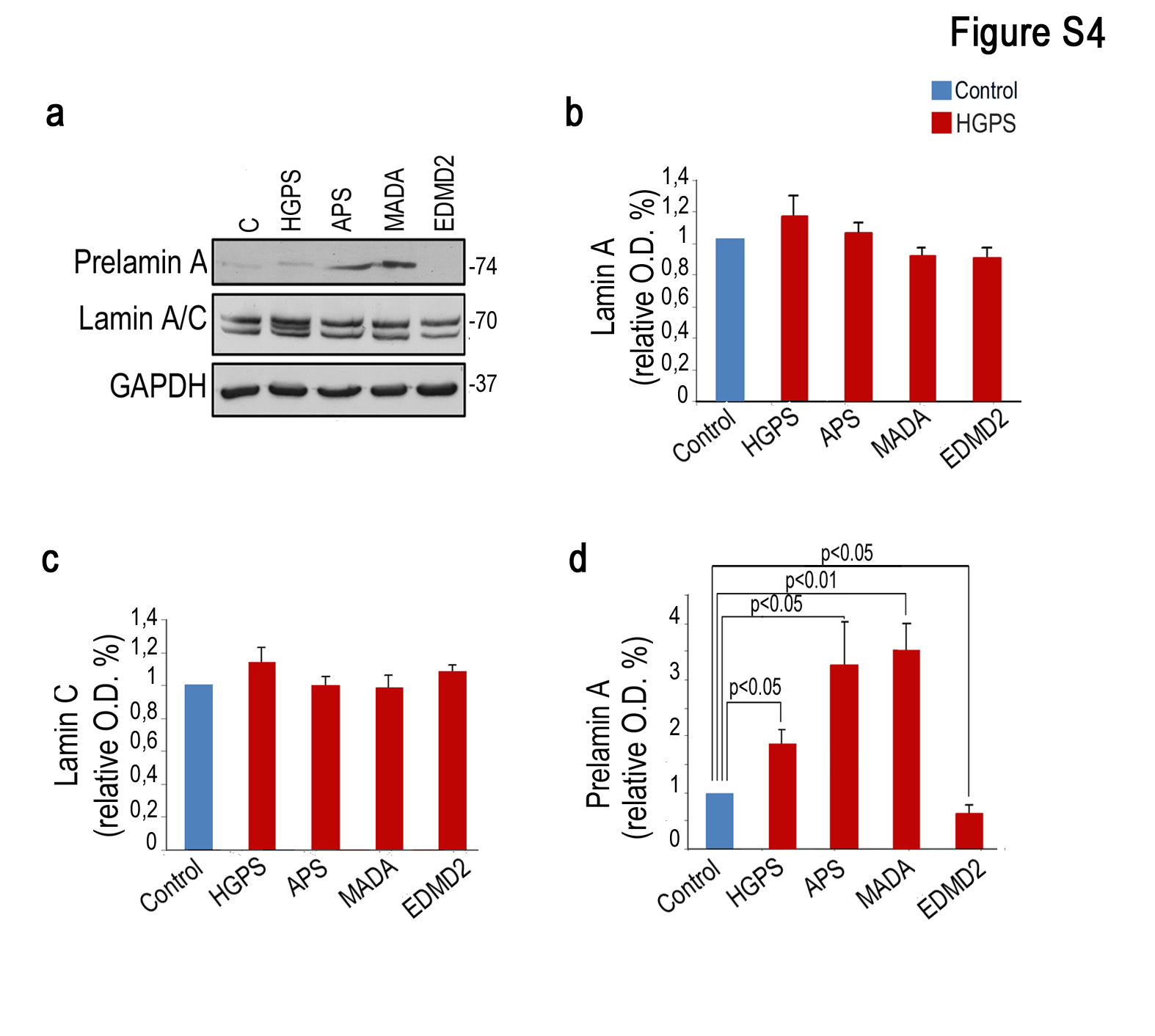

Supplement: Supplementary file 4 [file ACEL-17-e12824-s004.tif]

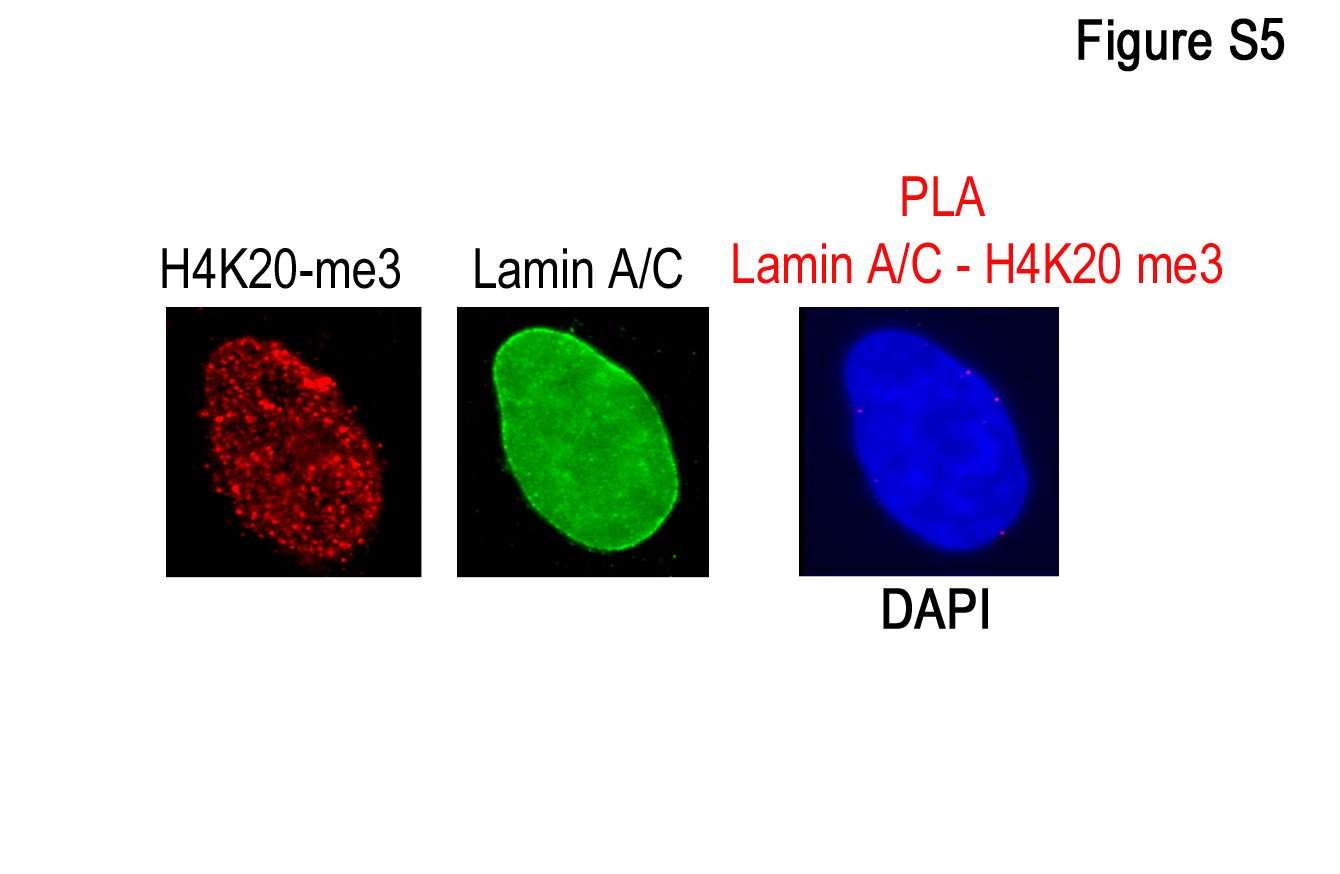

Supplement: Supplementary file 5 [file ACEL-17-e12824-s005.tif]

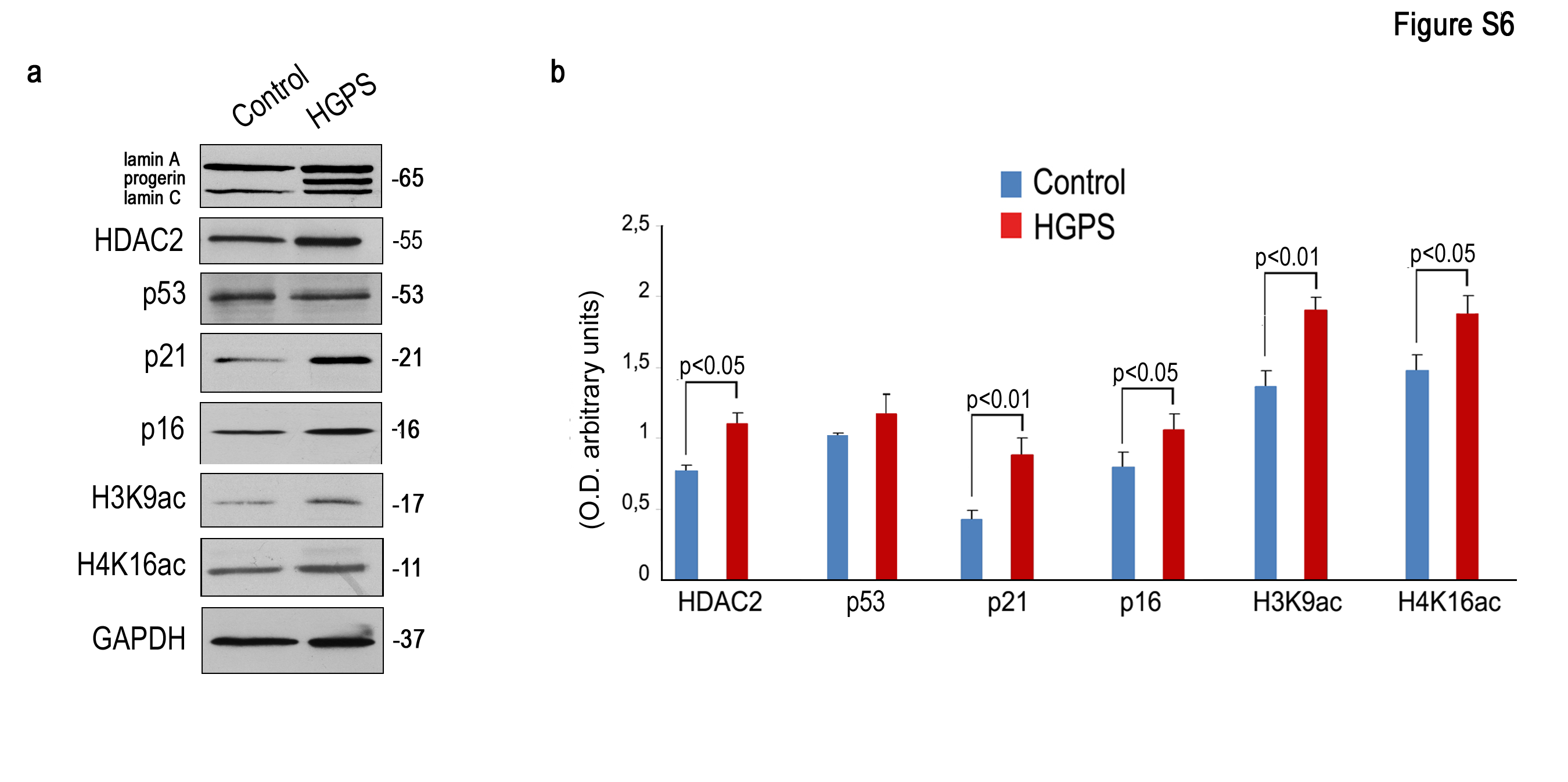

Supplement: Supplementary file 6 [file ACEL-17-e12824-s006.tif]
